# Single Nucleotide Polymorphisms on Toll-like Receptor-4 and the Risk of Developing Skin Cancer

**DOI:** 10.3390/ijms252312728

**Published:** 2024-11-27

**Authors:** Nabiha Yusuf, Noha Sharafeldin, Mohammad Saleem, Tom Callens, Ludwine M. Messiaen, Craig A. Elmets

**Affiliations:** 1 Department of Dermatology, University of Alabama at Birmingham, 1670 University Boulevard VH566A, Birmingham, AL 35294, USA; 2 Department of Hematology and Oncology, UAB Heersink School of Medicine, Birmingham, AL 35233, USA; 3 Medical Genomics Laboratory, Department of Clinical and Diagnostic Sciences, UAB School of Health Professions, Birmingham, AL 35233, USA

**Keywords:** toll-like receptor-4, single nucleotide polymorphisms, skin cancer

## Abstract

Exposure to solar ultraviolet (UV) radiation is an established risk factor for skin cancer. Toll-like receptor-4 (TLR4)-mediated immune dysregulation has emerged as a key mechanism for the detrimental effects of acute and chronic UV exposure and skin cancer in mice. Single nucleotide polymorphisms (SNPs) on the *TLR4* gene have been reported to increase or decrease susceptibility to various cancers in other organs. There is limited information on *TLR4* SNPs and susceptibility to human keratinocyte carcinomas. The study’s objective is to test the association between *TLR4* SNPs and the risk of developing keratinocyte carcinomas. Skin cancer patients and controls at the University of Alabama at Birmingham completed a cross-sectional survey on personal and family history of skin cancer as well as on sunscreen use and tanning proneness. Peripheral blood samples were obtained from participants, and DNA was extracted to genotype the *TLR4* SNPs. Descriptive analytics were used to describe the cohort. Multivariable logistic regression models were used to assess the association between *TLR4* SNPs and skin cancer risk. The sample consisted of a cohort of 93 skin cancer patients over the age of 50 and 94 controls; 33.3% of cases and 44.7% of controls were females; 12.9% of cases and 17% of controls had a *TLR4* SNP. The most common SNP was D299G/T399I in 9.7% of skin cancer patients and 13.8% of controls. We did not find a statistically significant association between the D299G/T399I SNP and skin cancer (odds ratio (OR) = 0.34, 95% CI: 0.11, 1.07, *p* = 0.065) adjusting for age, sex, eye color, actinic keratosis, sunscreen use and reapplication, and family history of skin cancer. Based on our findings from our limited cohort of participants, we found some protective effect for the *TLR4* SNP for skin cancer, which was not statistically significant. Validation of these findings in a larger cohort is warranted.

## 1. Introduction

Non-melanoma skin cancer (NMSC) is the most commonly diagnosed cancer in the United States (US). An estimated 1.8 million cases of squamous cell carcinoma (SCC) and 3.6 million cases of basal cell carcinoma (BCC) are diagnosed in the US each year [1], and $4.8 billion is expended on the management of NMSC cases [2]. NMSC cases also account for more than 5400 deaths every month worldwide [3]. Risk factors for NMSC include exposure to ultraviolet (UV) radiation; skin color; blue, hazel, and green eye color; a history of blistering sunburns; advanced age; immunosuppression; and a positive family history. Single nucleotide polymorphisms (SNPs) interfere with the function of some genes and influence the risk of skin cancer [4,5].

Toll-like receptor-4 (TLR4) belongs to a family of toll-like receptors that are involved in the innate immune response and can also lead to the activation of the adaptive immune response by maturation of dendritic cells, expression of co-stimulatory molecules, and promotion of Th1 responses [4]. TLR4 is expressed both on immune cells and cancer cells and thus acts as a double-edged sword. It can activate the host immune response against tumors but can also lead to tumor progression via its activation of inflammatory signaling cascades [6,7]. Studies from our laboratory have shown that the activation of TLR4 can have a protective effect on chemically induced skin tumors in mice. This effect was due to the activation of cell-mediated immune responses mediated by the chemical carcinogen 7,12-benz(a)anthracene [8]. On the other hand, we, and others, have shown that TLR4-mediated chronic inflammation can lead to tumor progression in a mouse model of ultraviolet (UV) B-mediated carcinogenesis [9]. Recently, we have observed that mice bearing the TLR4 D299G/T399I SNPs were resistant to UVB-induced immune suppression in the TLR4 gene; the most common SNP is an A–G substitution at nucleotide position +896, downstream of the cDNA start codon. This missense mutation leads to an amino acid substitution of Asp299Gly in the third exon of the TLR4 gene. This SNP was later shown to co-segregate with SNP Thr399Ile—also in the third exon of TLR4 [10]. Several studies have shown the impact of SNPs in TLR4 on cell functions and signaling mechanisms.

Various TLR4 SNPs have been associated with breast [11,12], prostate [13,14], colorectal [15,16,17], liver [18,19], lung [20,21], ovarian [22], cervical [23], nasopharyngeal [24], head and neck [25], and gastric cancer [26].

There is limited information available on the association between TLR4 SNPs and the risk of skin cancer. In a recent study, TLR4 SNPs D299G and T399I were found to be associated with the clinicopathologic features, progression, and survival of melanoma patients. In this study, we investigated the association between TLR4 SNPs D299G and T399I and the risk of developing skin cancer in a cross-sectional case–control study in patients seen at the Dermatology Clinic at the University of Alabama at Birmingham.

## 2. Results

The median age of cases was 69.2 (50.7–88.7) years, while that of controls was 60.8 (50.0–86.0) years. The median age of cases was significantly higher (*p* = 0.004) than the median age of controls. For gender, 66.7% of the cases and 55.3% of the controls were males; 79.6% of cases and 73.4% of controls had a light natural eye color (blue, green, or hazel). The prevalence of actinic keratoses was significantly higher (*p* < 0.001) in cases (53.8%) compared to controls (19.2%). There was no significant difference in the use of sunscreen between cases (77.4%) and controls (85.1%). Re-application of sunscreen was significantly higher (*p* = 0.015) in controls (41.5%) compared to cases (24.7%). More cases (47.3%) reported a family history of skin cancer in first-degree relatives compared to controls (35.1%). However, the difference was not significant (*p* = 0.07). The results for demographic and clinical characteristics have been listed in Table 1.

**Figure 1:**
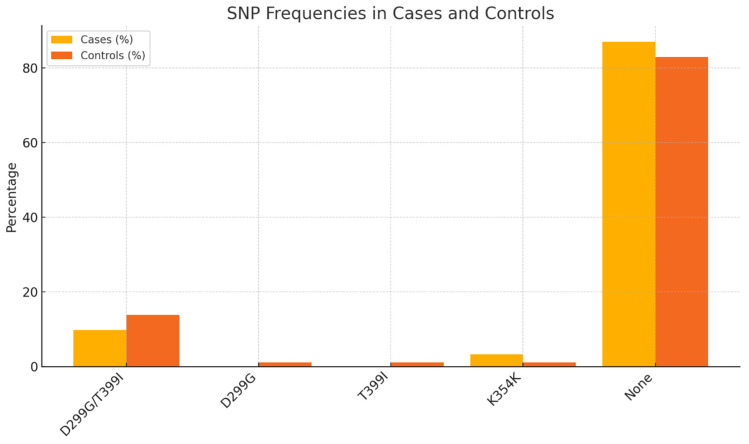
Frequency of TLR4 SNPs in cases and controls.

**Figure 2:**
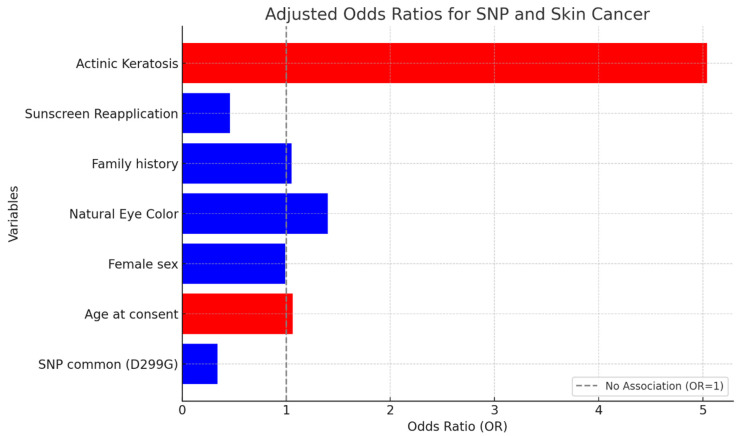
Adjusted odds ratio for association between TLR4 SNP and skin cancer.

A total of 187 subjects were typed successfully (93 skin cancer patients and 94 controls) for TLR4 D299G (A>G at the DNA level) and T399I (C>T at the DNA level) polymorphisms. The distribution of TLR4 (D299G and T399I) genotypes in cases and controls is presented in Table 1 and Figure 1. Only 12.9% of cases and 18% of controls had a *TLR4* SNP. The most common SNP was D299G/T399I in 9.7% of cases and 13.8% of controls with a TLR4 SNP. Single T399I and D299G SNPs were present in 1.1% of controls but were absent in all the cases. We also identified a previously unknown rare variant (i.e., K354K) that was covered by the T399I SNP and was present in 3.2% of cases and 1.1% of controls (Table 1, Figure 1). There was no significant association between the D299G/T399I SNP and skin cancer (odds ratio (OR) = 0.34, 95% CI: 0.11, 1.07, *p* = 0.065) adjusting for age at consent, sex, eye color, actinic keratosis, sunscreen use and reapplication, and family history of skin cancer (Table 2, Figure 2).

## 3. Discussion

In this study, we examined the association between TLR4 SNPs, mainly D299G and T399I, and the risk of non-melanoma skin cancer (NMSC) in a cohort from the University of Alabama at Birmingham. Our findings contribute to the limited information on how TLR4 SNPs can influence susceptibility to skin cancer.

TLR4 plays a crucial role in innate immunity and the activation of adaptive immune responses in several diseases, including cancer [4]. The role of TLR4 has been reported in several tumor types and has been implicated in both pro- and anti-tumorigenic processes [6,7,8]. Various TLR4 SNPs have also been reported to influence susceptibility or protection depending on the cancer type [11,12,13,14,15,16,17,18,19,20,21,22,23,24,25]. Our study adds to this body of knowledge by specifically examining the relationship between TLR4 SNPs and skin cancer risk.

The D299G/T399I polymorphism, which was the most common SNP observed in our study population, has been associated with altered TLR4 function and signaling. While our results suggest a protective effect of this SNP against skin cancer, the association did not reach statistical significance in our adjusted model. This finding contrasts with some previous studies that have reported increased cancer risk associated with this SNP in other cancer types [11,12,13,14,15,16,17,23]. However, it does align with studies that have found protective effects of TLR4 SNPs in certain cancers, such as hepatocellular carcinoma [18,19] and gastric cancer [26].

Our study also identified a previously unknown rare variant (K354K) in the TLR4 gene. While the functional implications of this variant are currently unknown, its presence in both cases and controls suggests that it may not have a significant impact on skin cancer risk. However, further investigation is needed to confirm this observation and explore any potential functional significance of this variant.

The complex role of TLR4 in skin carcinogenesis is further highlighted by previous studies using mouse models. Ahmad et al. demonstrated that TLR4 deficiency inhibited ultraviolet (UV) radiation-induced tumor development, suggesting a pro-tumorigenic role for TLR4 in this context [9]. Conversely, Yusuf et al. showed that TLR4 activation protects against chemically induced skin tumors in mice [8]. Our findings, which suggest a potential protective effect of TLR4 SNPs, align more closely with the latter study and underscore the nuanced and context-dependent role of TLR4 in skin cancer development. Our ongoing studies using a D299G/T399I TLR4 SNP mouse model have revealed a protective role of this SNP in UV-induced immune suppression, which is a major risk for skin cancer.

Our study also found significant associations between skin cancer risk and other factors such as age, the presence of actinic keratosis, and sunscreen reapplication habits. These findings reinforce the multifactorial nature of skin cancer development and the importance of considering both genetic and environmental factors in risk assessment.

TLR4 SNPs can interact with UV radiation to modulate skin immune responses through various mechanisms, thus influencing the outcome of UV exposure. It can alter the recognition of DAMPs by TLR4. TLR4 SNPs can also augment or dampen the cytokine response induced by TLR4 signaling. Alteration of TLR4 signaling due to the presence of SNPs can influence the DNA repair process and clearance of mutant cells, resulting in the progression or inhibition of skin tumor development. The exact impact depends on the nature of the SNP and its functional consequence on TLR signaling pathways. Understanding these interactions can inform therapeutic strategies to mitigate UV-related damage and associated disorders.

Our study has several limitations. First, the sample size was relatively small, which may have limited our ability to detect statistically significant associations, particularly for less common SNPs. Second, we used information on natural eye color as a proxy in our analysis. Third, we did not assess the functional consequences of the observed SNPs, which could provide valuable insights into the mechanisms underlying their potential protective effects.

Future studies should aim to replicate these findings in larger cohorts and explore the functional implications of TLR4 SNPs in skin cancer development. Additionally, investigating the interaction between TLR4 SNPs and environmental factors, such as UV exposure, and assessing the early biomarkers of cancer could provide a more comprehensive understanding of skin cancer risk.

In conclusion, our study suggests a potential protective effect of the TLR4 D299G/T399I SNP against skin cancer. The absence of the T399I SNP in cases and the identification of a novel K354K variant highlight the complex genetic landscape of TLR4 in relation to skin cancer risk. These findings contribute to our understanding of the role of TLR4 in skin carcinogenesis and may have implications for personalized risk assessment and prevention strategies in the future. However, further research is needed to fully elucidate the relationship between TLR4 genetic variations and skin cancer susceptibility.

## 4. Methods and Materials

### 4.1. Research Design

We performed a case–control study to investigate the association between TLR4 SNPs and the risk of skin cancer. We recruited males and females over the age of 50 with Fitzpatrick skin types I–IV. We recruited patients (N = 93) during their visit to our dermatologic surgery unit for skin cancer excision. Patients with tumor types other than basal cell carcinoma and squamous cell carcinoma, as well as the nevoid basal cell carcinoma syndrome, Cowden’s syndrome, xeroderma pigmentosum, or other syndromes with skin cancer predisposition, were excluded from the study. Patients with known exposure to arsenic or ionizing radiation, chronic immunosuppression due to organ transplant, anti-rejection regimen, or HIV/AIDS, and a history of prior treatment of tumor excised with immunomodulator therapy (imiquimod) were also excluded from the study. Controls were healthy individuals with no current or past history of skin cancer (N = 94). All the participants completed a questionnaire about their demographics, self-reported family history of skin cancer, and behavioral factors such as sunscreen use and reapplication. Peripheral blood (5 mL) was collected from each participant in an EDTA tube. All specimens were transported to the laboratory for further analysis. This study was approved (IRB# X080815001) by the Institutional Review Board of the University of Alabama at Birmingham and is registered with ClinicalTrials.gov (NCT03122366).

### 4.2. Methodologies

Genomic DNA was isolated from both case and control group blood specimens using a DNA isolation kit (Qiagen, Germantown, MD, USA), and a sequencing technique was employed for identifying the TLR4 SNPs, Asp299Gly (D299G) and Thr399Ile (T399I). The following primers were used for the D299G polymorphism (rs4986790): forward 5′-CCTGTGCAATTTGACCATTGAAG-3′ and reverse 5′-GAGAGATTTGAGTTTCAATGTGGG-3′, and for the T399I polymorphism (rs4986791): forward 5′-CGGATGGCAACATTAGAATTAGT-3′ and reverse 5′-CAGATGTTCTAGTTGTTCTAAGCCC-3′. For PCR amplification of both SNPs, a standard volume of gDNA (~25 ng/µL) per reaction was amplified by adding a master mix containing final concentrations of 1 µL 10xBuffer (BRL), 2 µL dNTPs (1 mM each), 0.32 µL MgCl_2_, 0.12 µL primer forward (50 µM), 0.12 µL primer forward (50 µM), 5.36 µL H_2_O, and 0.08 µL Platinum Taq (BRL) (5 units/µL). The PCR conditions for the D299G SNP were initial denaturation at 95 °C for 3 min, 8 cycles of denaturation for 20 s at 95 °C, and annealing for 15 s at 65 °C, followed by 30 min at 72 °C. This was followed by 25 cycles of 95 °C for 30 s, 58 °C for 30 s, and 72 °C for 30 s. The final cycle was performed at 72 °C for 10 s and 15 °C for 1 s. The PCR conditions for the T399I SNP were initial denaturation at 95 °C for 4 min, 41 cycles of denaturation for 30 s at 95 °C, and annealing for 30 s at 50 °C, followed by 30 min at 72 °C. The final cycle was performed at 72 °C for 10 s and 15 °C for 1 s. The PCR reactions were run in black 384-well fluorescence plates on a PCR machine, and sequencing was performed with the reverse primers using the ABI3130 (Applied Biosystems, Carlsbad, CA, USA). Sequencing for the TLR4 SNPs was performed at the Medical Genomics Laboratory at the University of Alabama at Birmingham.

### 4.3. Statistical Analysis

Clinical and demographic variables were summarized using the median (interquartile range) for continuous variables and the frequency (%) for categorical variables. Wilcoxon–Mann–Whitney tests were used for continuous variables, and Chi-square tests were used for categorical variables to detect statistically significant differences between the cases and controls. Associations with *p* < 0.05 were considered significant (all *p* values were two-tailed). Multivariable logistic regression was used for testing the association between the SNP and the case–control status adjusted for age, sex, natural eye color, family history of skin cancer, actinic keratosis, and sunscreen use and reapplication. All analyses were conducted using Stata14 (Stata, College Station, TX, USA).

## Figures and Tables

**Table 1:** Demographic and clinical characteristics of cases and controls.

Variables	Cases (n = 93)	Controls (n = 94)	*p* Value
Age at time of consent (years)			
Median (range)	69.2 (50.7–88.7)	60.8 (50.0–86.0)	<0.001
Sex, n (%)			
Females	31 (33.3%)	42 (44.7%)	0.16
Family history of skin cancer (first-degree), n (%)			
Yes	44 (47.3%)	33 (35.1%)	0.07
Natural eye color, n (%)			
Light (Blue/Green/Hazel)	74 (79.6%)	69 (73.4%)	0.32
Sunscreen use, n (%)			
Yes	72 (77.4%)	80 (85.1%)	0.29
Sunscreen reapplication, n (%)			
Yes	23 (24.7%)	39 (41.5%)	0.015
Actinic keratosis, n (%)			
Yes	50 (53.8%)	18 (19.2%)	<0.001
Common SNP			
D299G/T399I	9 (9.7%)	13 (13.8%)	
D299G	0 (0%)	1 (1.1%)	
T399I	0 (0%)	1 (1.1%)	
K354K	3 (3.2%)	1 (1.1%)	
None	81 (87.1%)	78 (83.0%)	0.44

**Table 2:** Adjusted odds ratios for the association between the SNP and skin cancer.

Variable	Odds Ratio (95% CI)	*p*-Value
SNP common (D299G)	0.34 (0.11–1.07)	0.065
Age at consent	1.06 (1.02–1.10)	0.004
Female sex	0.99 (0.46–2.13)	0.976
Natural eye color	1.40 (0.49–2.24)	0.418
Family history	1.05 (0.49–2.24)	0.909
Sunscreen use	0.89 (0.33–2.37)	0.814
Sunscreen reapplication	0.46 (0.21–1.03)	0.060
Actinic keratosis	5.04 (2.28–11.14)	<0.001

## Data Availability

The data presented in this study are available on request from the corresponding author.
